# Cimicifuga Racemosa

**Published:** 1861-07

**Authors:** 


ARTICLE III.

### CIMICIFUGA RACEMOSA.

### SIMPSON, PARRISH AND DAVIS.

[In looking over a recent file of the *Medical and Surgical Reporter*, (Feb. 16th, 1861,) our attention has been attracted by an article from the pen of Mr. EDWARD PARRISH, one of the most eminent of American Pharmaceutists; but in which he displays a want of familiarity with the literature of his profession, hard to account for on any hypothesis at all flattering to himself. If we mistake not, Prof. P. still holds his chair in the Philadelphia College of Pharmacy, and is honorably connected with the American Pharmaceutical Association, as well as with other home organizations. Yet in the course of his article, which is composed of therapeutical and pharmaceutical notes on *Cimicifuga*, occurs the following:

"The results, in this case, seem to indicate a trial of cimicifuga in anomalous cases of nervous disorder resisting ordinary stimulant and sedative treatment, and the many cases of chorea and rheumatism on record in which it has been found effectual, the testimony now coming across the water from the distinguished Edinburgh Professor, who has used it successfully in puerperal hypochondriasis, must draw increased attention to it as filling up a gap in the materia medica. Of the drug itself, too little is known by practitioners, who, in their daily rounds, pass by its nodding racemes projecting above the fence-tops, from Canada to Florida, without the least chagrin that some of its most valuable adaptations should be first brought to notice in a foreign land."

Of the merit and value of these strictures, the reader may easily satisfy himself, by a comparison of the subjoined, reprinted from the first volume of the *Transactions of the American Medical Association*, and the article of Prof. Simpson, among our *Selections*. Dr. Simpson will be found to have done American Pharmacy fuller justice than its Philadelphia representative, though he, by quoting an isolated sentence from the original report, makes the writer father an exaggeration, which exists only by this unintentional garbling. It will be seen, by a perusal of the Report, that the sentence quoted by Prof. Simpson, viz: "We have no more doubt of the efficacy of Cimicifuga in the early stages of acute rheumatism, than of the power of vaccination as a preventive of variola," is by no means the broad, sweeping assertion, with its context, that it seems isolated and unsustained.

What we had particularly in mind, however, in noticing this matter, is the lamentable tendency so prevalent in the profession to eagerly swallow any, the most exaggerated and monstrous, doctrines, inventions, remedies and *isms*, if they but come from "across the water." What divine virtue there is in a sea-voyage for a new fact or truth, we are unable to discern; but no one of our readers will be at a loss to call to mind more than one even ludicrous instance of this tendency to worship anything foreign—instances where, as in the present case, the matter originated here, had been transported to Great Britain or the Continent, translated, re-vamped and shipped back again, to be eagerly welcomed, re-translated and lauded by the very men, and those only, who had ignored its claims while it was yet untraveled. In this instance, it will be seen that not a single one of the adaptations of *Cimicifuga*, valuable or otherwise, was "first brought to notice in a foreign land;" and it is difficult to see how Prof. PARRISH could arrive at such a conclusion, with Prof. Simpson's article before him.—JUN. ED. EXAMINER.]

CIMICIFUGA RACEMOSA: *Actæa Racemosa. Black Cohosh.* Polypetalous exogenous. *Nat. Ord.* Ranunculacæa. *Sex. Syst.* Polyandria di Pentagynia.

The stem is simple, herbaceous, somewhat furrowed, from three to six feet high; flowers in a long terminal raceme with oftentimes one or more shorter ones at the base. The flowers are white, on short pedicles with small subulate bracts: calyx white, sepals four, rounded; petals small, shorter than the sepals, and cleft at their apex; stamens numerous with yellow anthers; pistil oval, with a lateral sessile stigma; the capsule is ovoid, dry, with one cell, containing many small flat seeds. The leaves are alternate, one nearly radical, large, decompound, and tripinnate; upper one bi-pinnate. Leaflets sessile, opposite, three to seven, dentate. The root is large, black, and fleshy, with numerous long, slender fibres. The root only is considered medicinal.

It has been used to a limited extent by the profession for many years; but its real properties are little understood. Indeed we are satisfied that the prevailing opinions in regard to its action on the system are entirely erroneous. Thus, we are told by Drs. Wood, Griffith, Lee, Williams, and others, that it is a stimulating tonic. By Dr. Chapman it is ranked among the expectorants; and by Dr. Martyn Payne in his recent Manual of Materia Medica, it is represented as a stimulant diaphoretic. In the United States Dispensatory, the almost universally received standard of authority in this country, we are told that the 'Cimicifuga unites with a tonic power, the property of stimulating the secretions, particularly those of the skin, kidneys, and pulmonary mucous membrane.' None of these opinions accord with our own experience in the use of this root; and we have used several pounds of it during the last few years, and in a considerable variety of diseases. We have never known it to produce a perceptible increase in any of the secretions of the system, nor has it the slightest stimulating qualities. But we have uniformly found it to lessen the frequency and force of the pulse, to soothe pain, and allay irritability. In a word, it is one of the most purely sedative agents we possess, making its impression chiefly on the nervous system of organic life. In large doses it produces vertigo, dimness of vision, and a depression of the pulse, which remain for a considerable time. These observations are directly confirmed by the experience of Dr. F. N. Johnson of this city, who has used it freely, both in his extensive private practice, and in the wards of the New York Hospital. He informs us that he has at different times selected more than twenty cases of acute inflammatory rheumatism, including the severest forms of that painful affection, and treated them with the Cimicifuga, for the purpose of fully testing its powers in that disease. The results were satisfactory in the highest degree, every vestige of the disease disappearing in from *two* to *eight* or *ten* days, without inducing any sensible evacuation, or leaving behind a single bad symptom. These trials have been repeated by Dr. Johnson, myself and others, until we have no more doubt of the efficacy of Cimicifuga in the early stages of acute rheumatism, than we have of the power of vaccination as a preventive of variola. Dr. Johnson found the most acute and severe cases that ever came under his observation, to yield to its influence, not only more speedily, but more perfectly and with less danger of metastasis to other organs, than to any other form of treatment.

The only visible effects of the medicine are diminution of the force and frequency of the pulse, disappearance of the arthritic pains and inflammation, with occasional vertigo or disposition to fall on attempting to assume the erect attitude. We place the more reliance on these observations of Dr. Johnson, not only on account of his deservedly high reputation as a practitioner, but because many of his trials were made in the wards of the hospital, where cases could be selected, and all the elements of comparison were much more perfect than in private practice. We are well aware that many other practitioners have used the Cimicifuga in rheumatism without the same happy results. But in every instance where we have had the opportunity to make the inquiry, we have found that they had mistaken the kind of cases, and the stage of the disease to which it is most applicable. From its being almost uniformly represented by medical writers as a stimulating excitor of the various secretions, the inference has been very naturally drawn, that it could only be applicable in the sub-acute and chronic forms of the disease, or at most, in the latter stages of the acute form. Whereas, in truth, these are precisely the class of cases in which it proves of comparatively little value; for its curative powers being dependent entirely on its *sedative* influence, exerted (as we believe), through the nerves of organic life, it can only prove effectual when given in the early stages, before the occurrence of those fibrinous deposits around the ligaments and parts affected, which so generally occur in the latter stages of protracted acute cases, and in all the more chronic forms of the disease. It may prove serviceable as an adjuvant to other remedies even in such cases, but it is only in the acute form of rheumatism that its own complete curative power is exhibited. And, indeed, we may say, as expressed to us by Dr. Johnson a few days since, that *the more acute the disease, the more prompt and decided will be the action of the remedy.* The properties we have attributed to the Cimicifuga, will readily suggest its applicability to the treatment of many other forms of disease besides rheumatism. And experience has already proved it valuable in some forms of chorea, hysteria, and other nervous affections.

The first paper concerning its effects in the treatment of chorea which has fallen under our notice, was from Dr. Young in the *American Journal of Medical Sciences*, vol. ix. p. 310, Feb., 1832. He there details several cases of this disease promptly and effectually cured by Cimicifuga alone. Since that time so many other similar cases have been reported by different writers, that we can no longer doubt its efficacy in this and kindred diseases when judiciously administered. We say judiciously, because it is not alike applicable to all the cases of any form of disease. Thus we may have chorea from the presence of worms or indigestible food, or other irritating matters in the alimentary canal, and no rational man ought to expect the cure of such cases by using a remedy that generally induces no sensible evacuations whatever. But in all those cases arising from undue irritability or mobility of the nervous system, a state so common in girls about the period when the menses ought to make their appearance, and which may be induced in both sexes by exposures to cold or other accidental influences, and even in those cases which may be kept up by what has been termed habit, after irritating matters that might have existed in the alimentary canal have been removed, we shall find the most decisive and successful results from the use of Cimicifuga in proper doses.

We recollect being called, about two years since, to see a young man several miles in the country, who was laboring under the most severe form of chorea that we ever saw. He was almost constantly in a state of irregular and severe muscular action; so much so that he could scarcely walk across the room, and sometimes so violent that it took two or three persons to keep him from injuring himself. The intervals were so short and imperfect that he scarcely got any sleep for weeks. A neighboring physician, under whose care he was, had tried all the ordinary remedies, including bleeding, blistering, emetics, cathartics, anodynes, antispasmodics, stimulants, and alteratives, both mineral and vegetable. On a careful examination I could not detect any local or inflammatory affection; almost the only indication of a morbid state consisting in a moderate increase of heat in the head. The case had plainly originated from a sudden exposure to cold while warm from hard labour. I directed the free use of a decoction of the Cimicifuga root, and the pouring of a stream of cold water over the occiput every hour until my next visit. Thirty-six hours after, I found him lying quietly in bed, considerably exhausted, and with a pulse not exceeding 60 per minute. The cold water was discontinued, the decoction given less freely; no return of the disease took place, and in a week or ten days the young man was again engaged in his labour. How much of the prompt and decisive effect was produced by the cold water, and how much by the Cimicifuga, the reader can judge for himself. We have no doubt but the influence of both was important, the first temporary, the latter permanent.

But it was in pulmonary affections, particularly in the early stages of Phthisis, that this remedy first attracted the notice of the profession. So early as 1823, Dr. J. S. Garden, of Charlotte, Va., published a paper in the *Medical Recorder*, in which he details its influence in his own case, as well as in several of his patients. In reference to his own case he says: " Shortly after commencing the use of this remedy, the hectic paroxysms, which had attended me some time previously, were entirely checked, the nocturnal evacuations from the surface of the body began to diminish, the expectoration of a fluid from the vessels of the lungs and bronchia, resembling pus in appearance, was speedily arrested; the cough became much less troublesome and less frequent. My pulse, which for some time before, was never lower than 100 to 120 pulsations to the minute, was reduced to the medium standard; the pain in my right breast and side left me, my strength and appetite began to improve, and I speedily abandoned the use of all medicines or other means, except attention to regimen and exercise. A period of twelve months or more had elapsed, from my primitive ill health to the time of using this medicine, during which time I had been bled freely and copiously, kept up a constant discharge from the surface of my breast by the use of blisters, setons, etc., and adhered strictly to a vegetable regimen, but without any relief." And in regard to the modus operandi of the medicine, he says: "It certainly possesses the power in an eminent degree of lessening arterial action, and at the same time imparting tone and energy to the general system.' In vol. V., new series, of the *American Journal of Medical Sciences*, 1843, the same writer again recurs to the use of this remedy, the utility of which only seems to have been confirmed by his subsequent experience of twenty years. In vol. IV. of the same journal we have an article from Dr. Charles C. Hildreth, of Zanesville, Ohio, on the use of the Cimicifuga in phthisis pulmonalis, in which he details three cases of this disease successfully treated by this remedy in conjuction with Iodine. He agrees fully with Dr. Garden, whose opinion in regard to its mode of action and effects we have already quoted. We have never relied on this remedy alone in treatment of any of the forms of pulmonary disease; but we have used it much in all the forms of tuberculous or scrofulous diseases, and with the most gratifying results, when combined with the preparations of Iodine. For some cases, and further observations on this point, we must refer the reader to an article in the *New York Journal of Medicine and Collateral Sciences*, vol. V. p. 314; premising, however, that since that article was written, our experience has satisfied us that the remedy is neither a stimulant or sensible promoter of the secretions, but a most valuable *sedative* in the true sense of that term. We have not been able to verify its reputed specific action on the uterus; though in many cases of severe headache, apparently depending on simple irritability of the brain in females of delicate habits, I have prescribed a decoction of the root in doses of a wineglassful every three or four hours, with the most prompt relief. Several attempts have been made by different individuals to analyze the root, without any very satisfactory results. It is certain, however, that it yields its specific virtues to alcohol more perfectly than to water. Hence it should generally be used in the form of saturated tincture, or in substance, pulverized. The first is prepared by macerating four ounces of the root in a pint of diluted alcohol, of which from thirty to sixty drops may be given every one, two, four, or six hours, according to the nature and severity of the case.

In acute rheumatism, from thirty to sixty gtts. of the tinct., or twenty grs. of the powdered root should be given to an adult every two hours, until its effects are manifest.

